# Pitx2 cholinergic interneurons are the source of C bouton synapses on brainstem motor neurons

**DOI:** 10.1038/s41598-019-39996-4

**Published:** 2019-03-20

**Authors:** Ismini Rozani, Georgia Tsapara, Emily C. Witts, S. James Deaville, Gareth B. Miles, Laskaro Zagoraiou

**Affiliations:** 1 Center of Basic Research, Biomedical Research Foundation of the Academy of Athens, 4 Soranou Ephessiou Str., 11527 Athens, Greece; 2 Division of Animal and Human Physiology, Department of Biology, National & Kapodistrian University of Athens, Panepistimiopolis, Ilisia, Greece; 3 Institute of Molecular Life Sciences, University of Zurich, Winterthurerstrasse 190, CH-8057 Zurich, Switzerland; 4 Sainsbury Wellcome Centre, 25 Howland Street, London, W1T 4JG UK; 5 School of Psychology & Neuroscience, University of St Andrews, Fife, KY169JP UK

## Abstract

Cholinergic neuromodulation has been described throughout the brain and has been implicated in various functions including attention, food intake and response to stress. Cholinergic modulation is also thought to be important for regulating motor systems, as revealed by studies of large cholinergic synapses on spinal motor neurons, called C boutons, which seem to control motor neuron excitability in a task-dependent manner. C boutons on spinal motor neurons stem from spinal interneurons that express the transcription factor Pitx2. C boutons have also been identified on the motor neurons of specific cranial nuclei. However, the source and roles of cranial C boutons are less clear. Previous studies suggest that they originate from Pitx2+ and Pitx2− neurons, in contrast to spinal cord C boutons that originate solely from Pitx2 neurons. Here, we address this controversy using mouse genetics, and demonstrate that brainstem C boutons are Pitx2+ derived. We also identify new Pitx2 populations and map the cholinergic Pitx2 neurons of the mouse brain. Taken together, our data present important new information about the anatomical organization of cholinergic systems which impact motor systems of the brainstem. These findings will enable further analyses of the specific roles of cholinergic modulation in motor control.

## Introduction

Freely moving animals face the challenge of organizing their muscular output to meet ever-changing environmental demands. Motor neurons that innervate the skeletal muscles are critical players in this process. Motor neurons must integrate a plethora of signals from the brain, the periphery, Central Pattern Generators and other local networks. Deciphering the circuits that provide tightly controlled input to motor neurons and regulate muscle output is imperative to further our understanding of neuromuscular physiology and pathology. One of these inputs is the cholinergic neuromodulation mediated by C bouton synapses^1–6^. In the spinal cord, these synapses facilitate task-dependent activation of motor neurons that control limb muscles. The source of C boutons on spinal motor neurons is cholinergic Pitx2 interneurons of the spinal cord^6^. Although C boutons have been well-studied within spinal motor circuits, much less is known about their roles within brainstem motor circuitry. In order to advance our understanding of the control of cranial motor neurons and, ultimately, the motor behaviors they control, we have focused on brainstem C boutons and the mapping of their putative sources.

C boutons are large (1–8 um in rodents^7^) cholinergic synapses on the somata and proximal dendrites of motor neurons^3,4,8,9^. They are characterized by sub-surface cisterns^1,10^, thin and elongated membrane vesicles in close apposition to the postsynaptic membrane at the synaptic cleft, detected by electron microscopy. The presence of choline acetyltransferase^8^ (ChAT) and vesicular acetylcholine transporter^11^ (vAChT) within C bouton terminals along with the postsynaptic expression of m2 muscarinic acetylcholine receptors, indicates that C bouton neurotransmission is cholinergic and metabotropic^12^. Synaptobrevin 2 (Vamp2) is also detected^13^ presynaptically, while Kv2.1 channels^14^, Ca^2+^ dependent K^+^ channels^15^, Sigma-1 receptors and neuregulin^16,17^ have been observed at the C bouton postsynaptic membrane. Previous studies^12,18,19^ have shown that C boutons are the only cholinergic synapses on the somata and proximal dendrites of motor neurons, while motor neuron collaterals form synapses in the distal dendrites, which are thought to be glutamatergic based on physiological evidence^20^. Insights into C bouton function have come from patch clamp experiments investigating the effects of activation of m2 muscarinic receptors on motor neurons. Activation of m2 receptors in spinal cord slices was found to increase motor neuron excitability, likely via a decrease in the after-hyperpolarization (AHP) potential^21,22^.

The source of spinal C boutons remained elusive for years after their initial description. The persistence of spinal C boutons following complete transection of the spinal cord^23^ and a lack of cholinergic projections from the brainstem to the thoracic and upper lumbar spinal cord^24^ suggested that spinal C boutons originate from spinal interneurons. In a transgenic ChAT-GFP mouse line (GENSAT), where the motor neurons, but not all cholinergic interneurons, were GFP+, C boutons were found to be GFP− and hence not derived from motor neurons, thus pointing to cholinergic partition cells as a putative source of C boutons^22^. Indeed, molecular genetics enabled us to identify the cholinergic subset (V0c) of a small interneuron population as the sole source of spinal C bouton synapses^6^. These interneurons express the transcription factor Pitx2 and form a rostrocaudal column along the spinal cord around the central canal. Inactivation of V0c cholinergic output, followed by intricate behavioral experiments, revealed an impairment in task-dependent changes in the activation of specific hind-limb muscles^6^. Thus, V0c interneurons and C boutons were shown to increase motor neuron output in a task-dependent manner.

C boutons are also present on motor neurons of some cranial motor nuclei. However, the cellular origins of brainstem C boutons and their roles in controlling motor output are less clear compared to their spinal counterparts. C boutons have been identified on brainstem motor neurons of rats and mice using immunohistochemical labelling and electron microscopy^3,4,8,11,12,25^. C boutons have been observed within specific branchiomotor and somatic nuclei, namely in the hypoglossal, trigeminal, facial nuclei and in the nucleus ambiguus^3,12,25–28^. On the contrary, C boutons and postsynaptic m2 receptors have been reported to be absent from oculomotor, trochlear, abducens nuclei and dorsal nucleus of vagus, a parasympathetic nucleus^12,29^.

A recent study investigated the origin of C bouton synapses on cranial motor nuclei (facial, trigeminal, hypoglossal, nucleus ambiguus), by injecting biotinylated dextran amine (BDA), as an anterograde tracer, in specific cholinergic regions of the reticular formation containing Pitx2 neurons and in regions without Pitx2 neurons^28^. This study reported the presence of brainstem Pitx2+ neurons only in the caudal medulla around the central canal and in the caudal intermediate reticular nucleus (IRt), a percentage of which were found to be cholinergic. Injections in these areas yielded large labelled cholinergic synapses within motor nuclei. Additionally, injections in regions that lack Pitx2 neurons including the rostral IRt, the ventral part of the medullary reticular nucleus (MdV) and in the gigantocellular reticular nucleus (Gi), also led to labelling of large cholinergic synapses in one or more of the four motor nuclei studied. The authors concluded that the source of C boutons on brainstem nuclei are both Pitx2+ and Pitx2− cholinergic neurons in the medulla. Since this observation contrasts with our previous findings that all C boutons contacting spinal motor neurons are Pitx2 derived, we sought to re-examine the origin of brainstem C boutons using molecular genetics.

In an attempt to locate Pitx2 cholinergic populations that could be potential sources of brainstem C boutons, we conducted a mapping study of Pitx2 neurons in the brain. As evidenced in the literature, Pitx2 expression in the embryonic brain has been observed in postmitotic neurons^30^ in the diencephalon, mesencephalon, zona limitans intrathalamica, rhombomere 1 and ventral spinal cord^31–33^. More specifically, in the developing mouse embryo three midbrain Pitx2 populations have previously been described: a dorsal GABAaergic population in the superior colliculus, a ventromedial population and a population of cells in the region of the red nucleus with transient Pitx2 expression^34^. In the postnatal Central Nervous System (CNS), Pitx2 expression can be observed in the anterior part of the brain: subthalamic nucleus, posterior hypothalamus, the zona incerta, the medial mammillary and supramammillary nucleus. In the midbrain Pitx2 neurons can be found in the red nucleus and the deep gray area of the superior colliculus^35^. In the hindbrain Pitx2 neurons are observed in the caudal medulla^28^. Lastly, there are also Pitx2 neurons in the spinal cord^6^.

In this study we use molecular genetic tools, similar to those that led to the discovery that cholinergic Pitx2 (V0c) interneurons are the sole source of C boutons in the spinal cord, to reveal the origin of C bouton synapses on motor neurons of the brainstem and to determine the precise location of cholinergic and non-cholinergic Pitx2 populations in the brain.

## Results

### Identification of Pitx2 derived cholinergic C boutons on cranial motor nuclei

We have previously shown that Pitx2 neurons are the sole source of C boutons in the spinal cord. We aimed to investigate whether this is true for brainstem motor neurons as well. To this end we examined the origin of C bouton synapses on motor neurons of brainstem motor nuclei, in postnatal day 25 (P25) mice, using the Pitx2::cre;Rosa.stop.tdTomato genetic scheme that enables the visualization of the somata, as well as processes and synapses of Pitx2 neurons. We detected C boutons and motor neuron somata using an antibody against ChAT that labels both structures. The anti-vAChT antibody we tested showed low penetration in vibratome sections and weak signal in the motor neuron somata. However, as has been reported previously in brainstem motor nuclei^27^, we confirmed in confocal images of the superficial layers of sections that vAChT and ChAT colocalize in the C boutons. C bouton identity was also verified by m2 immunoreactivity in close apposition to the cholinergic marker. We found that ChAT+ varicosities on the surface of motor neuron somata were almost always in close apposition to postsynaptic m2 immunoreactivity (m2 and ChAT colocalized at 364/368 ChAT+ boutons) with the very rare incidence of m2- ChAT+ structures consistent with cholinergic axons in passage. Thus, as indicated by previous studies^12,18,19^, our data support that all ChAT+ puncta on the somata of motor neurons are C boutons.

**Figure 1:**
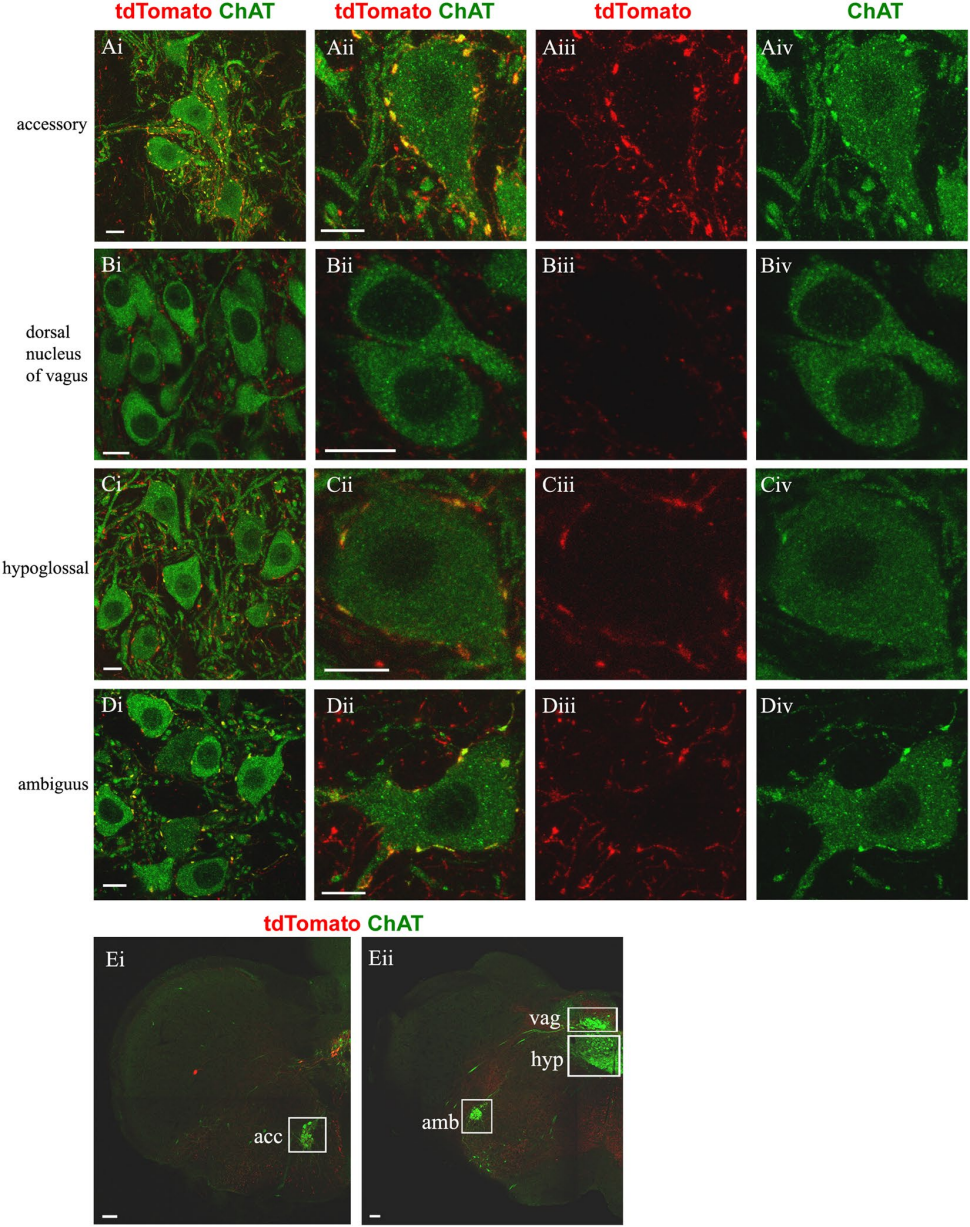
C boutons on brainstem motor neurons are Pitx2 derived. In green anti-ChAT antibody labels motor neuron somata and cholinergic C boutons on them, in red anti-dsRed antibody labels tdTomato+ Pitx2 derived synapses. (**A**–**D**) The first column contains images of the cranial motor nuclei and the next three columns contain close up images of motor neurons (merge, red only, green only). **(A**i–iv) Pitx2 derived (red) C boutons (green) on the accessory nucleus (green) appear yellow. (**B**i–iv) Dorsal nucleus of vagus does not receive C bouton inputs. (**C**i–iv**)** Pitx2 derived (red) C boutons (green) on the hypoglossal nucleus (green) appear yellow. (**D**i–iv**)** Pitx2 derived (red) C boutons (green) on the nucleus ambiguus (green) appear yellow. Scale bar (**A**–**D**) 10 μm. (**E**i) Half section of medulla showcasing the position of accessory nucleus, (**E**ii**)** dorsal nucleus of vagus, hypoglossal nucleus, nucleus ambiguus, in white boxes. Scale bar (**E**) 100 μm.

**Figure 2:**
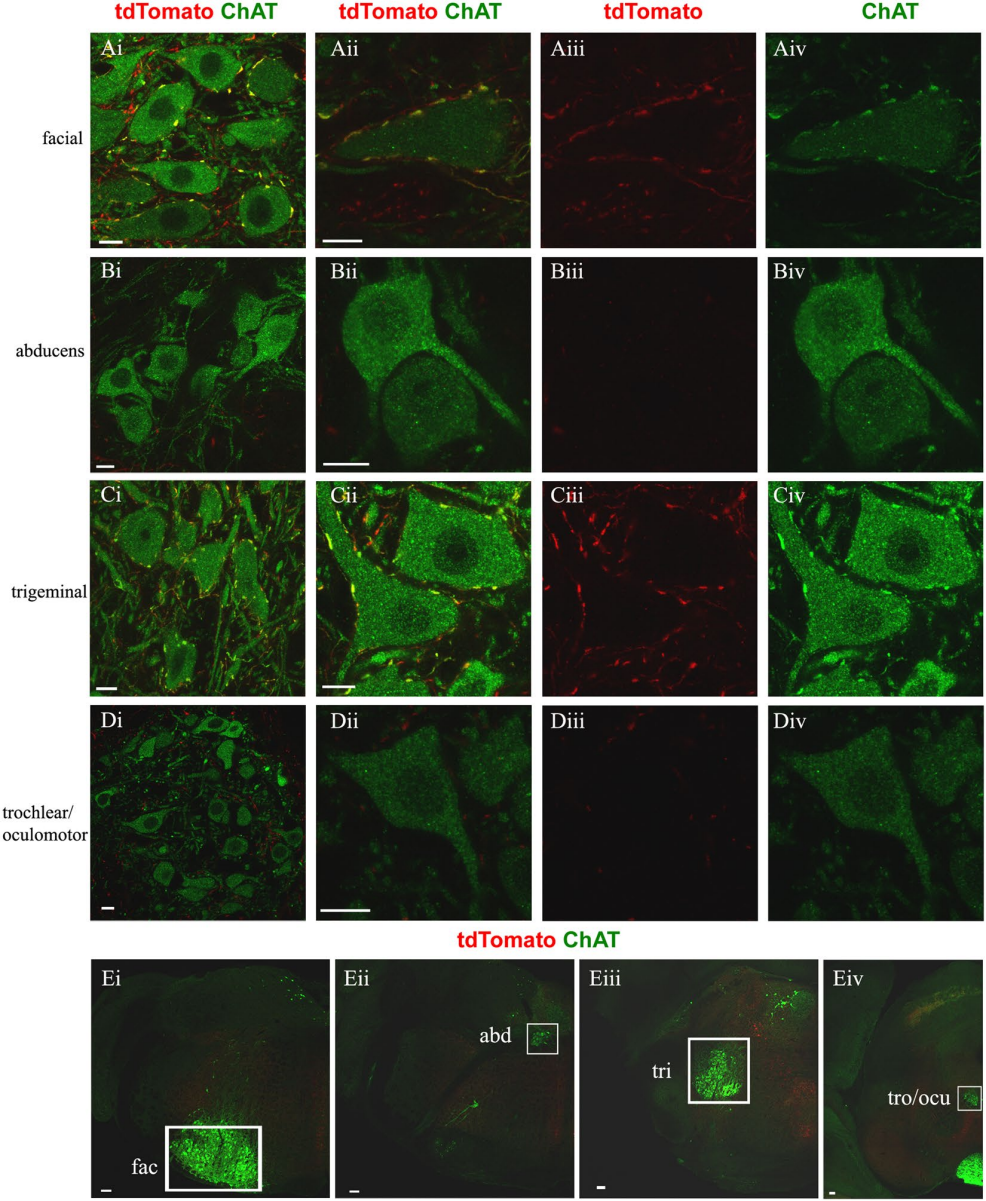
C boutons on brainstem motor neurons are Pitx2 derived. In green anti-ChAT antibody labels motor neuron somata and cholinergic C boutons on them, in red anti-dsRed antibody labels tdTomato+ Pitx2 derived synapses. (**A**–**D**) The first column contains images of the cranial motor nuclei and the next three columns contain close up images of motor neurons (merge, red only, green only). (**A**i–iv) Pitx2 derived (red) C boutons (green) on the facial nucleus (green) appear yellow. (**B**i–iv) Nucleus abducens does not receive C bouton inputs. (**C**i–iv) Pitx2 derived (red) C boutons (green) on the trigeminal nucleus (green) appear yellow. (**D**i–iv) Trochlear and oculomotor nuclei do not receive C bouton inputs. Scale bar (**A**–**D**) 10 μm. (**E**i) Half section of medulla showcasing the position of facial nucleus, (**E**ii**)** nucleus abducens, (**E**iii) trigeminal nucleus, (**E**iv) trochlear/oculomotor nucleus, in white boxes. Scale bar (**E**) 100 μm.

**Figure 3:**
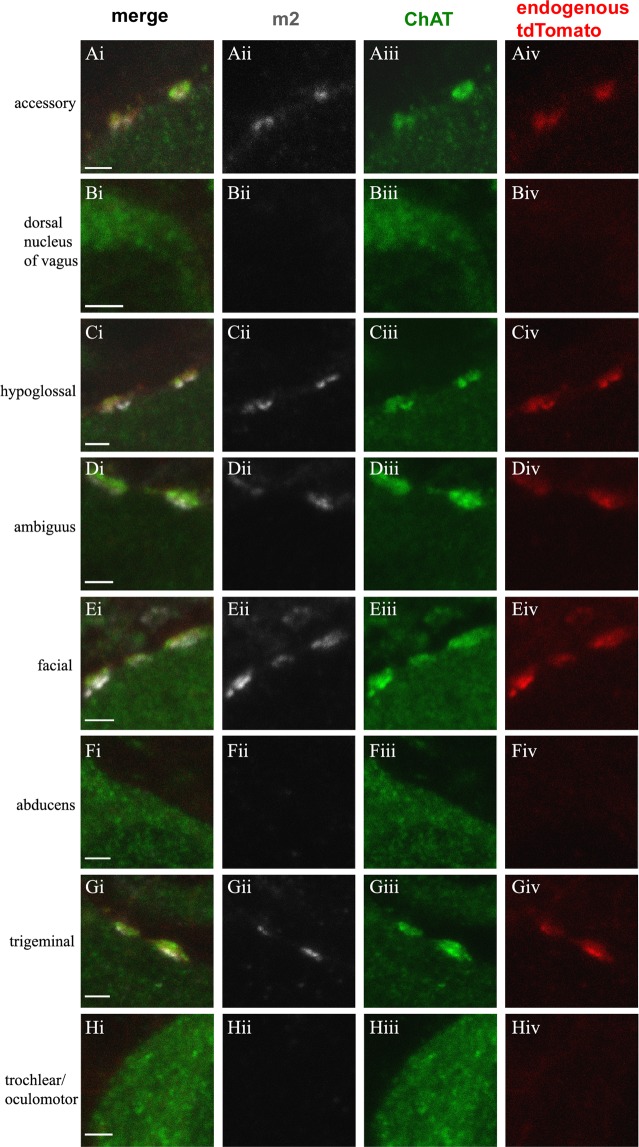
Verification of C bouton identity via postsynaptic m2 immunoreactivity. In white anti-m2 antibody, in green anti-ChAT antibody labels motor neuron somata and cholinergic C boutons on them, in red endogenous tdTomato+ fluorescence from Pitx2 derived synapses. (**A**) Pitx2 derived (red) C boutons (green) on the accessory nucleus (green) in alignment with m2 (white). (**B**) Dorsal nucleus of vagus lacks m2 immunoreactivity and C boutons. (**C**) Pitx2 derived (red) C boutons (green) on the hypoglossal nucleus (green) in alignment with m2 (white). **(D**) Pitx2 derived (red) C boutons (green) on the nucleus ambiguus (green) in alignment with m2 (white). (**E**) Pitx2 derived (red) C boutons (green) on the facial nucleus (green) in alignment with m2 (white). (**F**) Nuclus abducens lacks m2 immunoreactivity and C boutons. (**G**) Pitx2 derived (red) C boutons (green) on the trigeminal nucleus (green) in alignment with m2 (white). **(H**) Trochlear/oculomotor nucleus lacks m2 immunoreactivity and C boutons. Scale bar: 2 μm.

We next examined which non-autonomic cranial motor nuclei of the brainstem contain C boutons by performing labelling of hypoglossal, ambiguus, facial, abducens, trigeminal, and trochlear/oculomotor nuclei (these ocular nuclei were examined together as it is not possible to distinguish them in the mouse brain^36^). In addition to the above-mentioned motor nuclei, we also included in our analysis the accessory nucleus, located in the caudal medulla and the cervical spinal cord, and the dorsal nucleus of vagus, a parasympathetic nucleus, reportedly devoid of C boutons^12,37^, similarly to all neurons of the autonomic nervous system^38,39^. We observed large ChAT+ varicosities (Figs 1, 2), with postsynaptic m2 appositions on motor neurons (Fig. 3), which we defined as C boutons, within the accessory (Figs 1Ai–iv,Ei; 3Ai–iv), hypoglossal (Figs 1Ci–iv,Eii; 3Ci–iv), ambiguus (Figs 1Di–iv,Eii; Di–iv), facial (Figs 2Ai–iv,Ei; 3Ei–iv) and trigeminal (Figs 2Ci–iv,Eiii; 3Gi–iv) nuclei. C boutons were found to be absent from motor neurons of the dorsal nucleus of vagus (Figs 1Bi–iv,Eii; 3Bi–iv), nucleus abducens (Figs 2Bi–iv,Eii; 3Fi–iv), and trochlear/oculomotor nuclei (Figs 2Di–iv,Eiv; 3Hi–iv).

Having verified which motor nuclei contain C boutons, we next quantified the extent to which these brainstem C boutons are derived from Pitx2 neurons. To ensure that all tdTomato+ synapses were detected, we used an anti-DsRed antibody which also recognizes tdTomato. Dual immunostaining (ChAT, tdTomato) in tissue from Pitx2::cre;Rosa.stop.tdTomato mice enabled us to reveal that >98% C boutons were tdTomato+, and hence derived from Pitx2 neurons (Table 1). Taken together, these results support that, similarly to the spinal cord, Pitx2 neurons are the source of C bouton synapses on motor neurons of brainstem motor nuclei.

**Table 1:** Percentage of tdTomato+ C boutons on cranial motor neurons in Pitx2::cre;Rosa.stop.tdTomato mice.

	tdTomato+ ChAT+ C boutons/total ChAT+ C boutons									
	Accessory		Hypoglossal		Ambiguus		Facial		Trigeminal	
animal 1	100/100	100,00%	92/94	97,87%	73/73	100,00%	101/101	100,00%	141/141	100,00%
animal 2	276/277	99,64%	42/43	97,67%	47/49	95,92%	102/104	98,08%	198/200	99,00%
animal 3	—	—	105/106	99,06%	—	—	48/49	97,96%	172/172	100,00%
animal 4	172/173	99,42%	170/176	96,59%	63/63	100,00%	87/90	96,67%	62/62	100,00%
weighted average		99,63%		97,61%		98,90%		98,27%		98,37%
	**total: 98**,**58%**									

### Populations of Pitx2 neurons in the brain

We next investigated the location of Pitx2 neurons in the brain, with an emphasis on cholinergic cells, which represent potential cellular sources of brainstem C boutons. We again used the Pitx2::cre;Rosa.stop.tdTomato genetic scheme that labels neurons that have expressed the transcription factor Pitx2 at any time during development, along with immunohistochemical labelling for the cholinergic marker ChAT.

**Figure 4:**
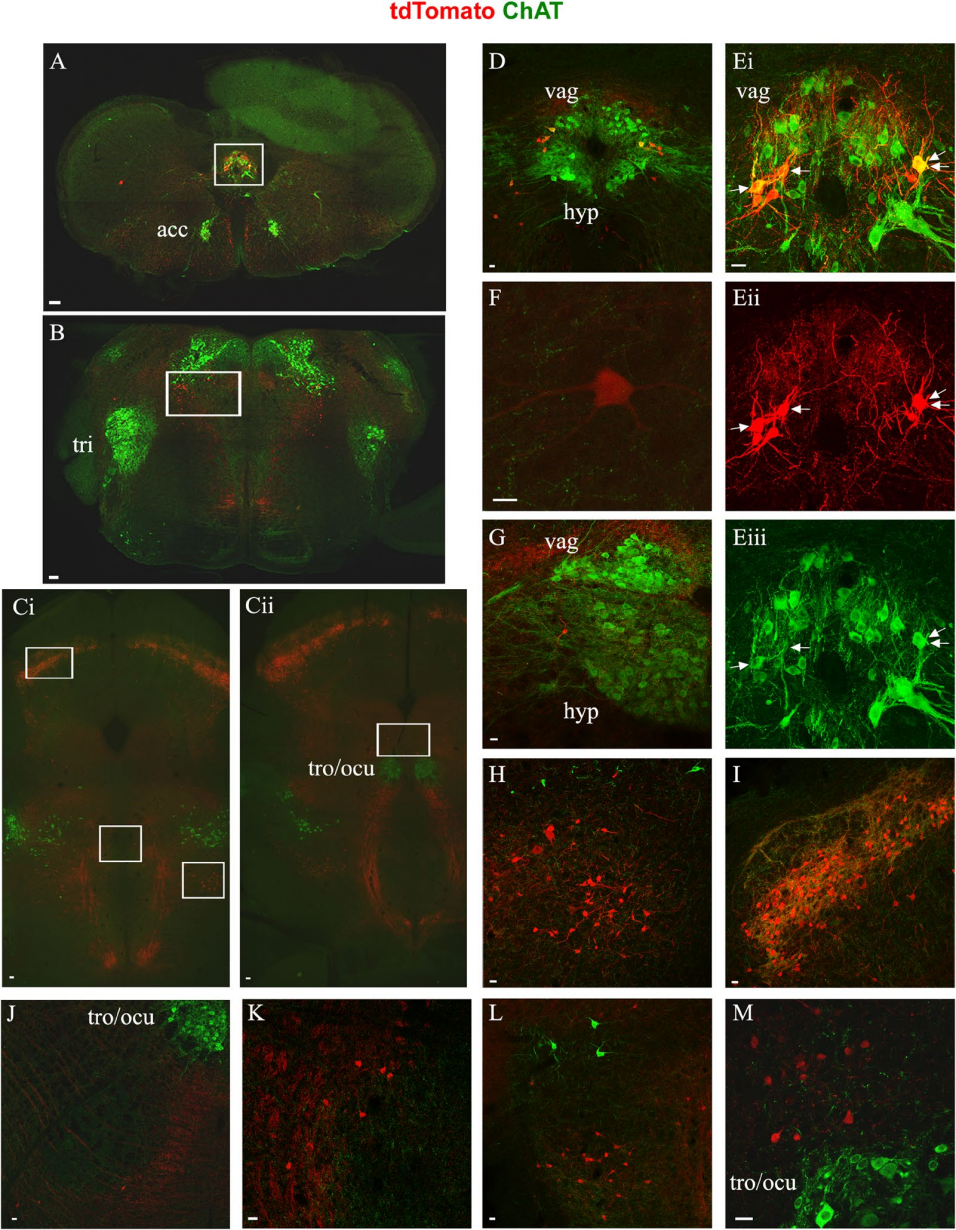
Pitx2 neurons of the brainstem. In green anti-ChAT antibody labels motor neuron somata, cholinergic interneurons and cholinergic synapses from interneurons and motor neurons, in red anti-dsRed antibody labels tdTomato+ Pitx2 neurons and Pitx2 derived synapses. Sections (**A**–**C**) presented in caudal to rostral succession (**A**) Caudal medulla section showcasing the localization of Pitx2 cholinergic and non-cholinergic neurons, in white box. (**B**) Pons section at the level of trigeminal nucleus. In red Pitx2 non-cholinergic neurons ventromedial and ventral of the laterodorsal tegmental nucleus indicated in white box. **(C**i,ii) Midbrain sections, i. in white boxes the populations of the superior colliculus, of the anterior tegmental nucleus and of the penduculopontine tegmental nucleus, ii. in white box the population in the supraoculomotor area. Scale bar (**A**–**C**) 100 μm. (**D**) Close up image of the caudal medulla Pitx2 neurons among the dorsal nucleus of vagus and the hypoglossal. (**E**i–iii) Close up image of caudal medulla cholinergic Pitx2 neurons, indicated with white arrows (i. merge, ii. red only, iii. green only). (**F**) Occasional non-cholinergic Pitx2 neurons appear in a lateral position away from the central canal. (**G**) The most rostral Pitx2 neurons in the medulla appear approximately in the middle of the dorsal nucleus of vagus and hypoglossal nucleus in the rostrocaudal axis. (**H**) Close up image of pons Pitx2 neurons. (**I**) Close up image of the Pitx2 neurons in the superior colliculus among cholinergic axons and synapses. (**J**) Close up of Pitx2 neurons in the red nucleus. (**K**) Close up of the Pitx2 neurons around the anterior tegmental nucleus (ATg). (**L**) Close up of Pitx2 neurons in the oral part of the pontine reticular nucleus (PnO), lateral to the tectospinal tract (ts) ventral to the penduculopontine tegmental nucleus (PPTg). (**M**) Close up image of Pitx2 neurons in the supraoculomotor area Scale bar (**D**–**K**) 20 μm.

**Figure 5:**
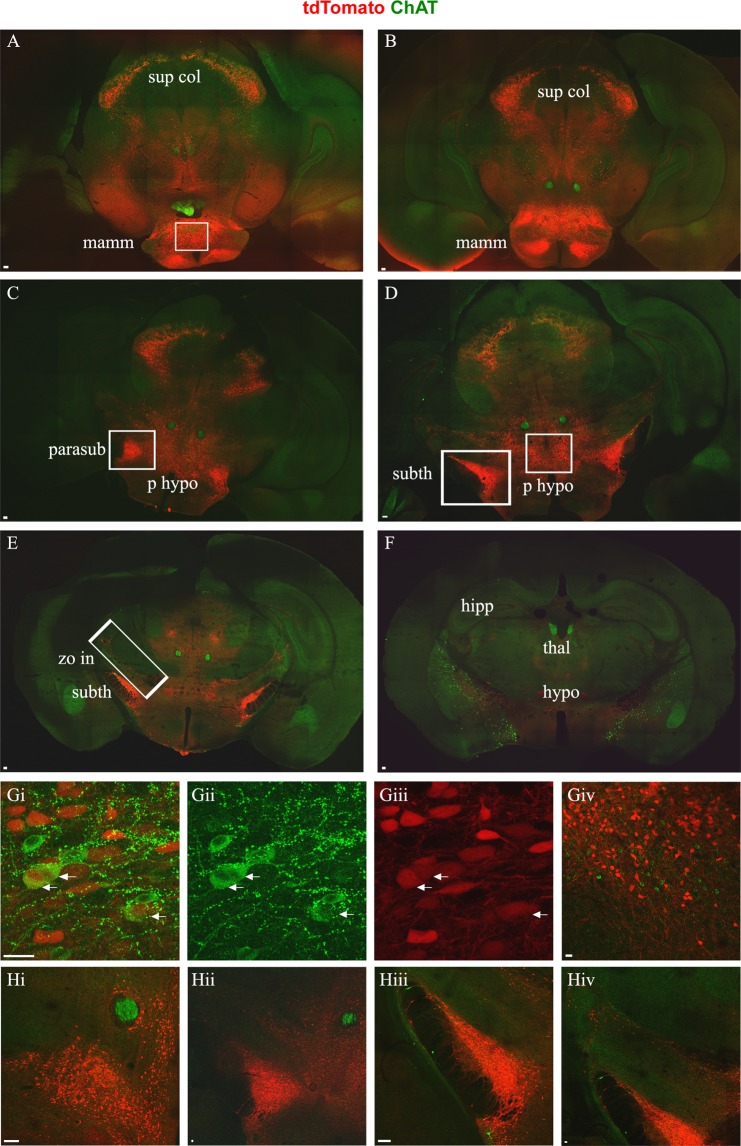
Pitx2 neurons of the anterior brain. In green anti-ChAT antibody labels motor neuron somata, cholinergic interneurons and cholinergic synapses from interneurons and motor neurons, in red anti-dsRed antibody labels tdTomato+ Pitx2 neurons and Pitx2 derived synapses. Sections (**A**–**F**) presented in caudal to rostral succession. (**A**) Pitx2 neurons in the mammillary area of the hypothalamus are present in the ventral part of the section (white box). The vast majority are non-cholinergic and very few at the boundary of supramammillary and medial mammillary nucleus are cholinergic. At the dorsal part of the section the midbrain Pitx2 neurons are still evident. (**B**) More rostral section with Pitx2 neurons in the mammillary area. (**C**) Pitx2 neurons evident in the posterior hypothalamus in the medial part of the section and the parasubthalamic nucleus at the lateral sides (white box). **(D**) At the lateral sides of the section, Pitx2 neurons appear in the subthalamic nucleus and in the medial area in the posterior hypothalamus (white boxes). (**E**) Subthalamic Pitx2 neurons and a line of Pitx2 neurons in the dorsal part of the zona incerta, dorsally to subthalamic nucleus (white box). (**F**) More rostral section that lacks Pitx2 somata, but few Pitx2 axons are still present. Scale bar (**A**–**F**) 100 μm. (**G**i–iii**)** Close up image of cholinergic mammillary Pitx2 neurons indicated with arrows (i: merge, ii: green only, iii: red only). (**G**iv) Close up image of the mammillary area with non-cholinergic Pitx2. (**H**i**)** Close up image of the non-cholinergic Pitx2 neurons in the posterior hypothalamus. (**H**ii**)** Close up image of the non-cholinergic Pitx2 neurons in the parasubthalamic nucleus. (**H**iii**)** Close up image of the non-cholinergic Pitx2 neurons in the subthalamic nucleus. (**H**iv**)** Close up image of the non-cholinergic Pitx2 neurons in the dorsal part of zona incerta, dorsally to the subthalamic nucleus. Scale bar (**G**,**H**) 20 μm.

In analysis of P25 mice, we observed tdTomato+ neurons, which will be referred to as Pitx2 neurons, in various areas of the brain: in the caudal medulla, the pons, the midbrain, and the hypothalamus (Figs 4, 5). In the caudal medulla, Pitx2 neurons appear to continue rostrally from the cervical spinal cord, as evidenced by their positioning around the central canal (Fig. 4A) up until regions of the medulla that coincide with approximately the middle of the hypoglossal nucleus (Fig. 4G). Some of the medullary Pitx2 neurons are positioned more ventrally in the region of the IRt and the MdV (Fig. 4D). Caudal medulla was the only region of the brainstem in which we observed cholinergic Pitx2 neurons (Fig. 4DEi–iii). Similarly to the spinal cord, these cholinergic neurons were intermingled with non-cholinergic Pitx2 neurons (Fig. 4A,D,Ei–iii). In addition, occasional non-cholinergic Pitx2 neurons appear in a lateral position away from the central canal (Fig. 4F).

The next group of Pitx2 neurons we observed was in the pons (Fig. 4B), beginning at the level of the trigeminal nucleus and continuing rostrally. With respect to neurotransmitter identity, all pons Pitx2 neurons were found to be ChAT−. These non-cholinergic pons Pitx2 neurons were located ventrally to the ChAT+ laterodorsal tegmental nucleus (LDTg) and dorsal tegmental nucleus (DTg) and medial to the trigeminal nucleus (Fig. 4H). They were dispersed throughout the dorsomedial tegmental area (DMTg) and possibly reached the dorsal part of the caudal pontine reticular nucleus (PnC) and the dorsal part of the subcoreuleus nucleus (SubCD).

We also observed non-cholinergic Pitx2 neurons in the following midbrain regions (Fig. 4Ci,ii): the first within layers of the superior colliculus, extending rostrally up to the intercollicular nucleus (InCo) and the deep mecencephalic region (DpMe) (Fig. 4Ci,ii,I), the second in the red nucleus (Fig. 4J), the third around the anterior tegmental nucleus (ATg) (Fig. 4Ci,K), the fourth in the oral part of the pontine reticular nucleus (PnO), lateral to the tectospinal tract (ts) ventral to the penduculopontine tegmental nucleus (PPTg) (Fig. 4Ci,L) and the fifth in the supraoculomotor area (Su3) (Fig. 4Cii,M). No colocalization of tdTomato and ChAT was observed in any of these midbrain regions.

Non-cholinergic Pitx2 neurons were also found to be present in more anterior brain regions (Fig. 5). This included the mammillary area, and in particular the supramammillary nucleus, the medial mammillary nucleus, and the premammillary nucleus (Fig. 5A,B). In contrast, the lateral mammillary nucleus lacked tdTomato+ neurons. Moving rostrally, Pitx2 neurons next appeared in and dorsal to the posterior hypothalamic nucleus (Fig. 5C,D,Hi), in the parasubthalamic nucleus (PSTh) of the lateral hypothalamus (Fig. 5C,Hii), in the subthalamic nucleus (Fig. 5D,E,Hiii) and in the dorsal part of zona incerta (Fig. 5D,E,Hiv). All of the aforementioned Pitx2 neurons were found to be non-cholinergic with the exception of a small number of cholinergic Pitx2 neurons located in a restricted area, among non-cholinergic Pitx2 neurons, at the boundary between the supramammillary nucleus and the mammillary nucleus, comprising less than 1% of the mammillary Pitx2 neurons (Fig. 5Gi–iv). In more rostral brain areas, including cortex and striatum, we did not detect Pitx2 neurons (Fig. 5F). In summary, cholinergic Pitx2 neurons in the CNS are located in the spinal cord, the caudal medulla and the mammillary area.

Given that the vast majority of C boutons are tdTomato+, we set out to calculate the recombination efficiency in cholinergic Pitx2 neurons. The recombination efficiency of the Rosa.stop.tdTomato reporter line was calculated at P5, as the anti-Pitx2 antibody detects the protein in a satisfactory manner until around P8 (Table 2). The recombination efficiency in cholinergic Pitx2 neurons of the cervical spinal cord is 88,77%, of the thoracic spinal cord (V0c) is 96,21%, while in the caudal medulla it was 78,21%. The recombination efficiency in the cholinergic Pitx2 neurons of the mammillary area was not calculated, because at P5 we could only identify rare cholinergic Pitx2 neurons in each section. Non-cholinergic Pitx2 neurons recombined with lower efficiency: in thoracic spinal cord the recombination rate was 62,92%, in cervical spinal cord 64,6%, while in the medulla 48,04%. Similarly, lower recombination rates in non-cholinergic Pitx2 neurons have been previously reported using the Pitx2::cre mouse line crossed with other reporter lines^6^. This could reflect lower activation of the *Pitx2* locus in non-cholinergic neurons. Overall, these data validate the use of this genetic model to map cholinergic Pitx2 neurons.

**Table 2:** Recombination efficiency in caudal medulla and spinal cord of Pitx2::cre;Rosa.stop.tdTomato mice.

	Cholinergic					
	tdTomato+ Pitx2+ Chat+ neurons/Pitx2+ Chat+ neurons					
	Medulla		Cervical spinal cord		Thoracic spinal cord	
animal 1	28/41	68,29%	51/56	91,07%	23/24	95,83%
animal 2	28/32	87,50%	28/33	84,85%	28/30	93,33%
animal 3	30/37	81,08%	—	—	25/25	100,00%
weighted average		78,21%		88,77%		96,21%
	**Non-cholinergic**					
	**tdTomato+ Pitx2+ Chat− neurons/Pitx2+ Chat− neurons**					
	**Medulla**		**Cervical spinal cord**		**Thoracic spinal cord**	
animal 1	111/242	45,87%	90/154	58,44%	30/47	63,83%
animal 2	101/194	52,06%	124/177	70,06%	40/59	67,80%
animal 3	89/193	46,11%	—	—	32/56	57,14%
weighted average		48,04%		64,60%		62,92%

In summary, our genetic labelling revealed a number of populations of Pitx2 neurons throughout the brainstem and brain. However, very few of these populations contained cholinergic neurons. In fact, cholinergic, Pitx2 neurons were only found in two areas: in the caudal medulla and a small population in the mammillary area of the hypothalamus. Thus, these two populations together with the spinal Pitx2 neurons represent the only cholinergic Pitx2 populations in the CNS.

## Discussion

Our understanding of the premotor circuits which control the output of cranial motor neurons varies: there is considerable knowledge of respiratory circuits located in the brainstem that regulate the inspiratory rhythm, while less is known about the premotor circuits controlling processes such as mastication, swallowing, head movements and facial expressions. Lessons from the local premotor circuits for locomotion indicate that neuromodulation is a critical player in the regulation of the excitation state of motor neurons and of their functional output. In order to shed light on the regulation of motor output and to provide an entry point for understanding diseases of impaired motor function, premotor circuits and neuromodulatory inputs affecting brainstem motor neurons call for more investigation.

In this study, we utilized approaches previously applied to the spinal cord to interrogate the origin of neuromodulatory cholinergic C bouton synapses on cranial motor neurons. C bouton synapses, a unique subtype of cholinergic synapse, are defined by their expression of cholinergic markers, such as ChAT or VAChT, by their size (1–8 um in rodents)^7^, by their localization on motor neurons in close opposition to subsurface cisterns and specific post-synaptic proteins, including m2 receptors and Kv2.1 channels^40^. In the present study, we chose to utilise an anti-ChAT antibody, because it marks both cholinergic synapses on motor neurons as well as cholinergic motor neuron somata.

In agreement with previous literature, we identified C boutons on motor neurons of the hypoglossal, ambiguus, trigeminal and facial motor nuclei (Figs 1; 2). Regarding nucleus ambiguus, a mixed somatic and visceral nucleus, large cholinergic synapses have been reported on only a subset of motor neuron somata, likely reflecting its dual functional roles^12,27,28,41^. Hellstrom *et al*.^12^ report that vAChT+ boutons on motor neurons of the nucleus ambiguus, are not colocalized with m2 or Vamp2, and therefore suggest that the nucleus ambiguus is devoid of C boutons. On the contrary, we observed large ChAT+ boutons that are m2+ and therefore identified as C boutons, on neurons of the nucleus ambiguus (Fig. 3Di–iv). This discrepancy might reflect the variability in motor neuron subtypes within the nucleus or may relate to differences in the level of magnification used for the identification of C boutons.

Out finding that >98% of brainstem C boutons originate from Pitx2+ neurons contrasts previous work which argued that C boutons only partially originate from Pitx2+ neurons^28^. Matsui and colleagues reached this conclusion based on experiments in which injections of anterograde tracer in the rostral medulla, an area lacking Pitx2 neurons, resulted in C bouton labelling in the facial, trigeminal and to a lesser extent ambiguus and hypoglossal motor nuclei. We hypothesize that this apparent discrepancy is likely to reflect uptake and diffusion of the dye by ascending axons, injured during the injection^42^, which originate from cholinergic Pitx2 neurons and pass through the rostral medulla to reach facial and trigeminal nuclei. The highest density of such axons would be in the vicinity of brainstem motor neurons with C boutons. Interestingly, the two injections yielding the most BDA labelled C boutons were inside the hypoglossal (caudal medulla) and the facial (rostral medulla) nuclei.

Evidence for the presence of premotor interneurons connected to cranial motor nuclei in the dorsal intermediate reticular nucleus (caudal medulla, at the level of the hypoglossal nucleus and the dorsal nucleus of vagus) and at the ventral intermediate reticular nucleus (rostral medulla, at the level of the nucleus ambiguus and facial nucleus) has been previously reported^43,44^. A small percentage of these premotor interneurons in both areas have been characterized as cholinergic^45,46^. Neurons in the caudal area are detected in a positon consistent with the one that we and others^28^ find the cholinergic Pitx2 neurons of the caudal medulla. The rostral area includes the regions injected by Matsui *et al*.^28^ that do not contain Pitx2 neurons but following injection led to C bouton labelling. We detected a small number of cholinergic Pitx2− neurons in the rostral medulla. We presume that either these neurons could send projections to distal dendrites of motor neurons or that they are not actually premotor interneurons. Given that previous studies have used retrograde labeling via injection into cranial motor nuclei to visualise premotor interneurons, it is possible that other neurons or axons in the proximity of the injection site have taken up the tracer, leading to false positive labeling. Indeed, there is evidence for cholinergic presynaptic inhibition of hypoglossal motor neurons^47^. Injury of these axons could result in labeling of cholinergic neurons that are not the source of C boutons. We believe that our use of mouse genetics for the identification of the origin of C boutons provides an additional, powerful tool which, taken together with our extremely high percentage of tdTomato+ C boutons, enhances the validity of our conclusion that brainstem C boutons do in fact arise from Pitx2 neurons.

Our results on the location of Pitx2 neurons in the brain parallel those in the literature on the adult rat brain^35^, with Pitx2 neurons revealed in the subthalamic nucleus, posterior hypothalamus, medial mammillary nucleus, supramammillary nucleus, red nucleus, and superior colliculus (Figs 4; 5). We also find Pitx2 neurons in the caudal medulla, at similar rostrocaudal levels to Matsui *et al*. and in the spinal cord. Regarding the Pitx2 population of the superior colliculus, there seems to be a controversy in the literature, that could be attributed to differences in the species and the developmental stage studied. Waite *et al*. suggest that Pitx2 neurons are located in the intermediate gray layer of the superior colliculus in the embryonic and postnatal mouse brain^34^, while Smidt *et al*.^35^, who studied the adult rat brain, reports that Pitx2 mRNA is present in the deep gray area of the superior colliculus. Waite *et al*. support their claim by using acetylcholinesterase detection to visualize the superficial layers and the intermediate gray area^48^. We have also noted dense cholinergic inputs around the Pitx2 neurons of the superior colliculus (Fig. 4I), therefore, in our hands, these neurons appear to be located in the intermediate gray area.

Apart from the aforementioned areas that have been previously reported in the literature, we report tdTomato+ neurons, in other areas of the brain where Pitx2 expressing cells have not previously been reported. In rostral parts of the brain we observe Pitx2 expression in the parasubtahlamic nucleus, while in the midbrain, expression was found in the oral part of the pontine reticular nucleus (PnO), in smaller numbers in the supraoculomotor area (Su3) and around the anterior tegmental nucleus (ATg) (Figs 4; 5). Although Pitx2 expression has not been previously reported in these areas, expression in the three midbrain areas may correspond to Pitx2 neurons described in the embryonic ventromedial midbrain^34^. More caudally, we have identified Pitx2 neurons in the pons at the level of the trigeminal nucleus. These neurons were not presented in the study by Matsui *et al*., where the authors searched for Pitx2 neurons in the brainstem, but they may relate to Pitx2 expression reported in rhombomere 1 in embryonic studies^32,49^. It is possible that we have observed more populations of Pitx2 neurons than previous studies in adult rodents^28,35^, because we identify them by the expression of tdTomato that persists after the downregulation of Pitx2 protein.

The neurotransmitter phenotype of the Pitx2 neurons has been studied in several of these populations. The subthalamic nucleus Pitx2 population is glutamatergic^50^, expressing vesicular glutamate transporter type 2^51^ and has received much attention due to the clinical implications of the subthalamic nucleus in the regulation of movement. The superior colliculus population has been identified as GABAergic and Pitx2 has been found to play an important role in their migration and GABAergic differentiation^34^. The pons Pitx2 neurons (rhombomere 1) are also GABAergic, but Pitx2 is not required for their differentiation^49^. Waite *et al*.^34^ show that in the embryonic mouse brain there are two ventral glutamatergic midbrain populations, a medial one and one in the red nucleus. Regarding the mammillary area, which is believed to be involved in spatial memory and vestibular processing, Pitx2 is critical for the formation of the mammillothalamic tract^52^. We show that the vast majority of mammillary Pitx2 neurons are non-cholinergic, with the exception of a very small number of cholinergic Pitx2 neurons, which comprise less than 1% of the total Pitx2 population in the mammillary area (Fig. 5Gi–iv) and are located mostly at the boundary between the medial mammillary nucleus and the supramammillary nucleus.

While C boutons contacting spinal motor neurons have a spinal origin, namely the cholinergic Pitx2 neurons surrounding the central canal of the spinal cord (V0c), the localization of the Pitx2 neurons that give rise to the brainstem C boutons is still unknown. Throughout the CNS only three cholinergic Pitx2 populations are detected: the spinal cholinergic, the caudal medulla cholinergic and the mammillary cholinergic Pitx2 neurons. Matsui *et al*. support a local origin as BDA injections in the caudal medulla result in labelling of C boutons in the motor nuclei examined. We found that almost all brainstem C boutons (>98%) are tdTomato+, and hence Pitx2 derived and one can hypothesize that the source cholinergic neurons would have comparable recombination efficiency. Spinal cord recombination efficiency in cervical cholinergic Pitx2 neurons is 88, 77%, in thoracic 96,21%, while the respective percentage for the caudal medullary cholinergic Pitx2 neurons is 78,21%. Thus, the spinal cord is a strong candidate source for brainstem C boutons. The recombination in caudal medulla is lower, but there is the possibility that the high Pitx2 expressing neurons (with higher possibility to recombine) are the source of C boutons. Although we cannot exclude the possibility that the mammillary cholinergic Pitx2 neurons contribute as well, the small mammillary cholinergic Pitx2 population is in a region known for their mammilothalamic and mammillotegmental projections and not for descending projections to medulla or involvement in motor control. Therefore, we favor a spinal, medullary or mixed origin. Should a neuromodulatory spinal to brainstem connection be proven, it could serve as a comodulator of cranial and spinal nuclei.

We showed that the C boutons in cranial motor nuclei originate solely from cholinergic Pitx2 neurons, similarly to the C boutons of the spinal cord (Figs 1; 2). Given the increase in excitability that Pitx2 neurons confer on spinal motor neurons via C bouton synapses, C boutons within cranial motor nuclei may modulate the excitability of brainstem motor neurons in a similar manner. The functional implications of C bouton activation in cranial motor nuclei have not yet been directly studied. However, muscarinic activation of the hypoglossal nucleus within *in vitro* preparations from neonatal rodents depolarizes neurons and enhances spike firing^21,53^, while in juvenile animals it leads to pre-synaptic inhibition of excitatory inputs^47^. Thus, there is clear evidence of metabotropic, cholinergic modulation of brainstem motor neurons, some of which might involve C boutons. Although there remains much to be determined regarding the functional roles of C boutons throughout brainstem motor nuclei, our data provide evidence of similarities in organization between spinal cord and cranial motor nuclei which may reflect similar development, organization and possibly regulation of all motor systems.

In summary, in this study we utilize molecular genetic tools to demonstrate that Pitx2 expressing neurons represent the sole source of C boutons contacting motor neurons within cranial motor nuclei including the accessory spinal nucleus, hypoglossal nucleus, nucleus ambiguus, facial nucleus and trigeminal nucleus. Moreover, by mapping Pitx2 neurons throughout the brain and brainstem we have identified new populations of Pitx2 neurons. Regarding cholinergic Pitx2 populations, there are three populations in the CNS, namely cholinergic Pitx2 neurons within the caudal medulla, cholinergic Pitx2 neurons of the spinal cord (V0c), and a small cholinergic mammillary Pitx2 population, that constitute the potential sources of cranial C boutons. Further investigation is needed in order to shed light on the relative contribution of these different populations to C bouton synapses on cranial motor nuclei and to determine whether there is any functional logic to their connectivity patterns.

## Methods

### Animals

Mice were housed in the animal facility of the Biomedical Research Foundation of the Academy of Athens or in the animal facility of the University of St. Andrews in rooms with a controlled light-dark cycle (12 h light–12 h dark) and free access to food and water. Pitx2::cre^54^ mice were crossed with the Rosa-stop-tdTomato^55^ reporter line. For the genotyping we used the following set of primers: 5′-GTCCAATTTACTGACCGTACACC-3′ and 5′-GTTATTCGGATCATCAGCTACACC-3′ for Pitx2::cre, 5′-GGCATTAAAGCAGCGTATCC-3′ and 5′-CTGTTCCTGTACGGCATGG-3′ for Rosa-stop-tdTomato. Male and female compound heterozygote progeny at postnatal day P4–6 (n = 3) and P24–26 (n = 6) were anesthetized and transcardially perfused.

### Vibratome sectioning

After transcardial perfusion with ice cold PBS, the mice were perfused with fresh 4% PFA. The brain was removed and fixed in 4% PFA overnight at 4 °C. After 2hrs washes with PBS, the brain was embedded in 4% agarose and 50um coronal vibratome sections were obtained on ice (Leica VT1000S).

### Immunohistochemistry and imaging

Sections were incubated in 0.5% Triton-X100 in PBS for 10 min and then incubated in a humidified chamber with the primary antibodies in blocking solution (1% BSA, 0.3% Triton-X100 in PBS) at 4 °C for 48 hrs, while rocking. After 5 × 60 min washes in PBS, sections were incubated in a humidified chamber with the primary antibodies in antibody solution (1% BSA, 0.3% Triton-X100 in PBS) at 4 °C overnight, while rocking. Following 8 × 30 min washes in PBS, sections were mounted on Polysine (Thermo Scientific) slides with Vectashield (Vector) and then covered. All images were obtained using confocal laser scanning microscopy (LeicaTC S SP5 II), except Fig. 4Ci,Cii that were obtained using fluorescent microscope. The low magnification confocal images are tiles stitched together using ImageJ. Primary antibodies used: rabbit anti-dsRed (1:2000, Clontech), rabbit anti-m2 (1:500, Alomone), goat anti-ChAT (1:400 for vibratome sections, 1:100 for cryosections, Millipore), rabbit anti-Pitx2 (1:16000, described in Zagoraiou, 2009), guinea pig anti-vAChT (1:6000, Fitzerald). Anti-rabbit Cy3, anti-rabbit Alexa 488, anti-goat Alexa 488, anti-goat Cy5, anti-guinea pig 488 (1:1000 for Cy3 and 1:500 for the Alexa 488 and Cy5, Jackson Immunoresearch) secondary antibodies were diluted in blocking solution.

tdTomato+ C boutons (tdTomato+ ChAT+ boutons/ChAT+ boutons) on cranial motor nuclei were counted in 4 mice (P24–26), from confocal images of vibratome sections (4–7 sections per nucleus per mouse) throughout the rostrocaudal axis of each nucleus.

m2 identity of ChAT+ bouton was confirmed in 2 mice (P24–26) from confocal images of vibratome sections (3–5 sections per nucleus per mouse) throughout the rostrocaudal axis of each nucleus as m2+ ChAT+/ChAT+ boutons.

Analysis of the Pitx2 populations was conducted in 6 mice (P24–26).

Recombination efficiency was calculated, as the weighted average of the percentage in question (i.e. ChAT+ tdTomato+ Pitx2+ neurons over total ChAT+ Pitx2+ neurons), in 3 mice (P4–6) from confocal images of cryosections (19–26 sections per mouse for the caudal medulla, 36–38 sections per mouse for cervical spinal cord and 13 sections per mouse for thoracic spinal cord.

The Mouse Brain in Stereotaxic Coordinates, by Paxinos and Franklin was used as a reference for the recognition of anatomical brain structures. The Allen brain atlas (*in situ* hybridization data) was used as a reference of the brain areas that contain Pitx2 neurons.

### Ethical Approval

The study was partially performed in the Laboratory Animal Unit of Centre of the Biomedical Research Foundation of the Academy of Athens, in accordance to the European legal framework for the protection of animals used for scientific purposes (European Convention 123/Council of Europe and European Directive 2010/63/EU), the National law in harmonization to the above mentioned European Directive as well as the current Guidelines of International Organizations such as the Association for the Assessment and Accreditation of Laboratory Animal Care International-AAALAC Int., and the Federation of European Laboratory Animal Science Associations-FELASA. The competent Regional Veterinary authority approved the experimental protocol in accordance to Greek legislation (Presidential Decree 56/2013, in compliance with the European Directive 2010/63).

For work conducted in St Andrews, all procedures performed on animals were conducted in accordance with the UK Animals (Scientific Procedures) Act 1986 and were approved by the University of St Andrews Animal Welfare and Ethics Committee.

Abbreviations used in the images for the cranial motor nuclei: acc: accessory, vag: dorsal nucleus of vagus, hyp: hypoglossal, amb: ambiguus, fac: facial, abd: abducens, tri: trigeminal, tro/ocu: trochlear/ocumotor, sup col: superior colliculus, mamm: mammillary area, parasub: parasubthalamic nucleus, p hypo: posterior hypothalamus, subth: subthalamic nucleus, zo in: zona incerta, hipp: hippocampus, thal: thalamus, hypo: hypothalamus.

## Data Availability

The datasets generated and analysed during the current study are available from the corresponding author on reasonable request.

## References

[1] Conradi S, Skoglund S (1969). Observations on the ultrastructure of the initial motor axon segment and dorsal root boutons on the motoneurons in the lumbosacral spinal cord of the cat during postnatal development. Acta Physiol. Scand. Suppl..

[2] Barber RP (1984). The morphology and distribution of neurons containing choline acetyltransferase in the adult rat spinal cord: an immunocytochemical study. J. Comp. Neurol..

[3] Nagy JI, Yamamoto T, Jordan LM (1993). Evidence for the cholinergic nature of C-terminals associated with subsurface cisterns in alpha-motoneurons of rat. Synap. N. Y. N.

[4] Li W, Ochalski PA, Brimijoin S, Jordan LM, Nagy JI (1995). C-terminals on motoneurons: electron microscope localization of cholinergic markers in adult rats and antibody-induced depletion in neonates. Neuroscience.

[5] Wilson JM, Rempel J, Brownstone RM (2004). Postnatal development of cholinergic synapses on mouse spinal motoneurons. J. Comp. Neurol..

[6] Zagoraiou L (2009). A cluster of cholinergic premotor interneurons modulates mouse locomotor activity. Neuron.

[7] Alvarez FJ, Dewey DE, McMillin P, Fyffe RE (1999). Distribution of cholinergic contacts on Renshaw cells in the rat spinal cord: a light microscopic study. J. Physiol..

[8] Connaughton M, Priestley JV, Sofroniew MV, Eckenstein F, Cuello AC (1986). Inputs to motoneurones in the hypoglossal nucleus of the rat: light and electron microscopic immunocytochemistry for choline acetyltransferase, substance P and enkephalins using monoclonal antibodies. Neuroscience.

[9] Arvidsson U, Riedl M, Elde R, Meister B (1997). Vesicular acetylcholine transporter (VAChT) protein: a novel and unique marker for cholinergic neurons in the central and peripheral nervous systems. J. Comp. Neurol..

[10] Conradi S (1969). Ultrastructure and distribution of neuronal and glial elements on the motoneuron surface in the lumbosacral spinal cord of the adult cat. Acta Physiol. Scand. Suppl..

[11] Gilmor ML (1996). Expression of the putative vesicular acetylcholine transporter in rat brain and localization in cholinergic synaptic vesicles. J. Neurosci. Off. J. Soc. Neurosci..

[12] Hellström J, Oliveira ALR, Meister B, Cullheim S (2003). Large cholinergic nerve terminals on subsets of motoneurons and their relation to muscarinic receptor type 2. J. Comp. Neurol..

[13] Hellström J, Arvidsson U, Elde R, Cullheim S, Meister B (1999). Differential expression of nerve terminal protein isoforms in VAChT-containing varicosities of the spinal cord ventral horn. J. Comp. Neurol..

[14] Muennich EA, Fyffe RE (2004). Focal aggregation of voltage-gated, Kv2.1 subunit-containing, potassium channels at synaptic sites in rat spinal motoneurones. J. Physiol..

[15] Deardorff AS (2013). Expression of postsynaptic Ca2+ −activated K+ (SK) channels at C-bouton synapses in mammalian lumbar -motoneurons. J. Physiol..

[16] Mavlyutov TA (2012). Development of the sigma-1 receptor in C-terminals of motoneurons and colocalization with the N,N′-dimethyltryptamine forming enzyme, indole-N-methyl transferase. Neuroscience.

[17] Gallart-Palau X (2014). Neuregulin-1 is concentrated in the postsynaptic subsurface cistern of C-bouton inputs to α-motoneurons and altered during motoneuron diseases. FASEB J. Off. Publ. Fed. Am. Soc. Exp. Biol..

[18] Cullheim S, Kellerth JO, Conradi S (1977). Evidence for direct synaptic interconnections between cat spinal alpha-motoneurons via the recurrent axon collaterals: a morphological study using intracellular injection of horseradish peroxidase. Brain Res..

[19] Lagerbäck PA, Ronnevi LO, Cullheim S, Kellerth JO (1981). An ultrastructural study of the synaptic contacts of alpha-motoneurone axon collaterals. I. Contacts in lamina IX and with identified alpha-motoneurone dendrites in lamina VII. Brain Res..

[20] Bhumbra GS, Beato M (2018). Recurrent excitation between motoneurones propagates across segments and is purely glutamatergic. PLOS Biol..

[21] Lape R, Nistri A (2000). Current and voltage clamp studies of the spike medium afterhyperpolarization of hypoglossal motoneurons in a rat brain stem slice preparation. J. Neurophysiol..

[22] Miles GB, Hartley R, Todd AJ, Brownstone RM (2007). Spinal cholinergic interneurons regulate the excitability of motoneurons during locomotion. Proc. Natl. Acad. Sci. USA.

[23] McLaughlin BJ (1972). Propriospinal and supraspinal projections to the motor nuclei in the cat spinal cord. J. Comp. Neurol..

[24] VanderHorst VG, Ulfhake B (2006). The organization of the brainstem and spinal cord of the mouse: relationships between monoaminergic, cholinergic, and spinal projection systems. J. Chem. Neuroanat..

[25] Davidoff MS, Irintchev AP (1986). Acetylcholinesterase activity and type C synapses in the hypoglossal, facial and spinal-cord motor nuclei of rats. An electron-microscope study. Histochemistry.

[26] Ichikawa T, Hirata Y (1990). Organization of choline acetyltransferase-containing structures in the cranial nerve motor nuclei and lamina IX of the cervical spinal cord of the rat. J. Hirnforsch..

[27] Ichikawa T, Ajiki K, Matsuura J, Misawa H (1997). Localization of two cholinergic markers, choline acetyltransferase and vesicular acetylcholine transporter in the central nervous system of the rat: *in situ* hybridization histochemistry and immunohistochemistry. J. Chem. Neuroanat..

[28] Matsui T (2013). C-terminals in the mouse branchiomotor nuclei originate from the magnocellular reticular formation. Neurosci. Lett..

[29] Vilaró MT, Wiederhold K-H, Palacios JM, Mengod G (1992). Muscarinic M2 receptor mRNA expression and receptor binding in cholinergic and non-cholinergic cells in the rat brain: A correlative study using *in situ* hybridization histochemistry and receptor autoradiography. Neuroscience.

[30] Martin DM, Skidmore JM, Fox SE, Gage PJ, Camper SA (2002). Pitx2 distinguishes subtypes of terminally differentiated neurons in the developing mouse neuroepithelium. Dev. Biol..

[31] Kitamura K, Miura H, Yanazawa M, Miyashita T, Kato K (1997). Expression patterns of Brx1 (Rieg gene), Sonic hedgehog, Nkx2.2, Dlx1 and Arx during zona limitans intrathalamica and embryonic ventral lateral geniculate nuclear formation. Mech. Dev..

[32] Muccielli ML, Martinez S, Pattyn A, Goridis C, Brunet JF (1996). Otlx2, an Otx-related homeobox gene expressed in the pituitary gland and in a restricted pattern in the forebrain. Mol. Cell. Neurosci..

[33] Lindberg C, Wunderlich M, Ratliff J, Dinsmore J, Jacoby DB (1998). Regulated expression of the homeobox gene, rPtx2, in the developing rat. Brain Res. Dev. Brain Res..

[34] Waite MR, Skidmore JM, Billi AC, Martin JF, Martin DM (2011). GABAergic and glutamatergic identities of developing midbrain Pitx2 neurons. Dev. Dyn. Off. Publ. Am. Assoc. Anat..

[35] Smidt MP (2000). Analysis of three Ptx2 splice variants on transcriptional activity and differential expression pattern in the brain. J. Neurochem..

[36] Sturrock RR (1991). Stability of motor neuron number in the oculomotor and trochlear nuclei of the ageing mouse brain. J. Anat..

[37] Ichikawa T, Shimizu T (1998). Organization of choline acetyltransferase-containing structures in the cranial nerve motor nuclei and spinal cord of the monkey. Brain Res..

[38] Mawe GM, Bresnahan JC, Beattie MS (1986). A light and electron microscopic analysis of the sacral parasympathetic nucleus after labelling primary afferent and efferent elements with HRP. J. Comp. Neurol..

[39] Leedy MG, Bresnahan JC, Mawe GM, Beattie MS (1988). Differences in synaptic inputs to preganglionic neurons in the dorsal and lateral band subdivisions of the cat sacral parasympathetic nucleus. J. Comp. Neurol..

[40] Deardorff, A. S., Romer, S. H., Sonner, P. M. & Fyffe, R. E. W. Swimming against the tide: investigations of the C-bouton synapse. *Front*. *Neural Circuits* **8** (2014). 10.3389/fncir.2014.00106 PMC4167003 25278842

[41] Bautista TG, Sun Q-J, Zhao W-J, Pilowsky PM (2010). Cholinergic inputs to laryngeal motoneurons functionally identified *in vivo* in rat: a combined electrophysiological and microscopic study. J. Comp. Neurol..

[42] Reiner A (2000). Pathway tracing using biotinylated dextran amines. J. Neurosci. Methods.

[43] Dauvergne C, Pinganaud G, Buisseret P, Buisseret-Delmas C, Zerari-Mailly F (2001). Reticular premotor neurons projecting to both facial and hypoglossal nuclei receive trigeminal afferents in rats. Neurosci. Lett..

[44] Popratiloff AS (2001). Hypoglossal and reticular interneurons involved in oro-facial coordination in the rat. J. Comp. Neurol..

[45] Travers JB, Yoo J-E, Chandran R, Herman K, Travers SP (2005). Neurotransmitter phenotypes of intermediate zone reticular formation projections to the motor trigeminal and hypoglossal nuclei in the rat. J. Comp. Neurol..

[46] Volgin DV, Rukhadze I, Kubin L (2008). Hypoglossal premotor neurons of the intermediate medullary reticular region express cholinergic markers. J. Appl. Physiol. Bethesda Md 1985.

[47] Bellingham MC, Berger AJ (1996). Presynaptic depression of excitatory synaptic inputs to rat hypoglossal motoneurons by muscarinic M2 receptors. J. Neurophysiol..

[48] Beninato M, Spencer RF (1986). A cholinergic projection to the rat superior colliculus demonstrated by retrograde transport of horseradish peroxidase and choline acetyltransferase immunohistochemistry. J. Comp. Neurol..

[49] Waite MR (2012). Distinct populations of GABAergic neurons in mouse rhombomere 1 express but do not require the homeodomain transcription factor PITX2. Mol. Cell. Neurosci..

[50] Martin DM (2004). PITX2 is required for normal development of neurons in the mouse subthalamic nucleus and midbrain. Dev. Biol..

[51] Schweizer N (2014). Limiting glutamate transmission in a Vglut2-expressing subpopulation of the subthalamic nucleus is sufficient to cause hyperlocomotion. Proc. Natl. Acad. Sci..

[52] Skidmore JM, Waite MR, Alvarez-Bolado G, Puelles L, Martin DM (2012). A novel TaulacZ allele reveals a requirement for Pitx2 in formation of the mammillothalamic tract. Genes. N. Y. N 2000.

[53] Bellingham MC, Funk GD (2000). Cholinergic modulation of respiratory brain-stem neurons and its function in sleep-wake state determination. Clin. Exp. Pharmacol. Physiol..

[54] Liu W, Selever J, Lu M-F, Martin JF (2003). Genetic dissection of Pitx2 in craniofacial development uncovers new functions in branchial arch morphogenesis, late aspects of tooth morphogenesis and cell migration. Dev. Camb. Engl..

[55] Madisen L (2010). A robust and high-throughput Cre reporting and characterization system for the whole mouse brain. Nat. Neurosci..

